# The Diagnostic Yield of Colonoscopy Stratified by Indications

**DOI:** 10.1155/2017/4910143

**Published:** 2017-07-27

**Authors:** I. Al-Najami, C. P. Rancinger, Morten Kobaek Larsen, E. Spolén, G. Baatrup

**Affiliations:** ^1^Department of Surgery, Odense University Hospital and Svendborg Hospital, Valdemarsgade 53, 5700 Svendborg, Denmark; ^2^Department of Clinical Research, University of Southern Denmark, Odense, Denmark

## Abstract

**Introduction:**

Danish centers reserve longer time for screening colonoscopies and allocate the most experienced endoscopists to these cases. The objective of this study is to determine the diagnostic yield in colonoscopies for different indications to improve planning of colonoscopy activity and allocation of the highly skilled endoscopists.

**Methods:**

Nine hundred and ninety-nine randomly collected patients from a prospectively maintained database were grouped in defined referral indication groups. Five groups were compared in respect of the detection rate of adenomas and cancers.

**Results:**

Two hundred and eighty-nine of 1098 colonoscopies in 999 patients showed significant neoplastic findings, resulting in 591 adenoma resections. Eighty-five percent were treated with a snare resection, and 15% with endoscopic mucosa resection (EMR). Positive findings in the indication groups were (1) symptoms, 25%; (2) positive screening, 17%; (3) previous resection of adenomas, 45%; (4) previous resection of colorectal cancer, 15%; and (5) surveillance of patients with high-risk family history of cancer, 35%.

**Conclusion:**

The majority of adenomas found during colonoscopy can be treated with simple techniques. If individualized time slots are considered, the adenoma follow-up colonoscopies are likely to be the most time-consuming group with more than twice the number of adenomas detected as compared to other indications.

## 1. Introduction

Introduction of the National Screening Program has increased the demand for colonoscopy capacity by approximately 25% [1]. The centers performing colonoscopies are challenged by the increased workload as experienced in the UK and The Netherlands [2]. This may lead to longer waiting times for the symptomatic patients and to additional expenses for the hospitals [3]. The need for advanced endoscopic procedures has increased more than correspondingly [4]. Few dedicated physicians master those, and in the majority of the Danish endoscopy units, this leads to referral for a second therapeutic colonoscopy by EMR, ESD, or transanal endoscopic microsurgery (TEM) for the removal of advanced adenomas [5]. Thus, a booking strategy for a colonoscopy based upon indications may be useful to allocate the right patients to experienced endoscopists and/or reserve individualized time slots for patients based upon the a priori risk of positive findings. Further, the instructions in Denmark as well as in the UK advise the units to allocate the most experienced endoscopists to screening colonoscopies, without any supporting evidence (Danish Colorectal Cancer Group, DCCG Guidelines, August 2014, http://dccg.dk/retningslinjer/august2014/2014_screening.pdf, cited 2016 June) [6].

The most common indications for colonoscopies are unexplained anemia, hematochezia, diarrhea, and a positive screening test [7, 8]. However, the diagnostic yield depends on the indication, with unexplained diarrhea and blood in the stools having a high diagnostic yield [9] along with the detection of occult blood in the stool [10, 11].

Available data regarding the prevalence, clinical features, and significance of a colonoscopy in the evaluation of colorectal polyps is widely published [12, 13], but an investigation of the diagnostic yield of a colonoscopy for different indications is needed. The objective of our study was to investigate the adenoma and cancer detection rates stratified by different indications.

## 2. Methods

The enrollment of 999 patients was made by random extractions from a prospectively maintained database, according to 5 defined indications for referral during the period from September 1, 2013 to June 31, 2015, from our unit. The indications were the following: (1) symptoms, (2) positive iFOBT screening test, (3) follow-up after earlier resection of adenomas, (4) follow-up after segmental bowel resection for colorectal cancer, and (5) surveillance of patients with high-risk family history of cancer (HNPCC or FAP).

Data were crosschecked with the local regional patient data registry and the local electronic patient files. Patients were randomly selected from the database, from a list of patients who were referred to a colonoscopy at the surgical department. All indications were related to the increased risk of colorectal adenomas and cancer. Patients referred to a colonoscopy due to symptoms had one or more of the following symptoms: rectal bleeding or visible blood in stools, abdominal discomfort or changed bowel habits for more than 2 months, unintended weight loss, or anemia. A positive colorectal screening test was based on microscopic blood findings in the stool obtained by the immunological FOBT method according to Danish guidelines with a cutoff value of 100 *μ*g/L; 209 of the 251 patients were first-time attenders for a colonoscopy. A follow-up after treatment for benign colorectal adenomas included only endoscopically resected tumors. The follow-up was offered to patients with high-risk histological features, severe dysplasia, tumors larger than 10 mm, or more than 3 adenomas resected at the same colonoscopy. All the resected adenomas were judged macroscopically radically resected by the endoscopist and microradically resected by the pathologist with a 100% agreement level. All of the resected adenomas had a full colonoscopy at the index colonoscopy; the time from the index colonoscopy to the follow-up colonoscopy was minimum 12 months for those resected by EMR and 1 year for those resected by a simple snare technique if they were high risk and 3 years if they were low risk. The follow-up after treatment for a malignant tumor was offered after 1 year and every third year for patients treated with a bowel resection. Patients included in the surveillance were diagnosed with familial adenomatous polyposis, Peutz-Jeghers syndrome, and hereditary nonpolyposis colorectal cancer or had at least one first-degree relative diagnosed with one of these conditions. They underwent a colonoscopy every third year. Inclusion of at least 250 colonoscopies was accomplished for all indications except for surveillance of patients with a family history of colorectal cancer. We were able to include 96 colonoscopies in this group only. A total of 999 patients to achieve a minimum of 1000 colonoscopies were enrolled; a total of 1098 colonoscopies were enrolled (Table 1).

Each patient was registered once only and could only be allocated to 1 indication group.

All adenomas were included regardless of size and histology. No serrated lesions were included in this study.

General approval by the Danish Data Protection Agency and approval by the local ethics committee of Southern Denmark (registration number: s-20140075/2008-58-0035) were obtained. The database is registered as a quality assessment database and is therefore not registered in clinicaltrials.gov or other freely registered databases.

## 3. Results

### 3.1. Demographics

Nine hundred and ninety-nine patients with 1098 colonoscopies were enrolled, 497 were female, and 601 were male, with the mean age of 64.5 and 66.7, respectively. Of the total number of colonoscopies, 289 had significant neoplastic findings, resulting in 591 resected adenomas, 84% of which with a simple snare technique and 16% with EMR or ESD during a second procedure (Table 1).

### 3.2. Indications and Diagnostic Yield

#### 3.2.1. Symptoms

Approximately, one fourth (25.2%, *n* = 63/250) of the colonoscopies performed for symptoms revealed one or more polyps. The total number of adenomas found was 117. The mean number of adenomas per patient was 1.86 (*n* = 117/63). Forty-seven patients were treated with a simple snare resection, 17 were referred to an advanced local resection, and 2 cancers were detected. Four patients (6%, *n* = 4/63) had a history of previous benign adenomas. In 187/250 (74.8%) colonoscopies, no adenomas were found (Table 2).

#### 3.2.2. Positive Screening

Seventeen percent (*n* = 42/251) of the patients having a colonoscopy preceded by a positive screening test had adenomas, 38 of them were resected with a snare, 10 were referred to advanced endoscopic resection, and 3 cancers were detected (Table 2).

#### 3.2.3. Adenoma Follow-Up

Almost half of the patients previously treated for adenomas had adenomas at follow-up (45%, *n* = 113/250). Seventy-six percent were previously treated with a simple snare resection, and 21% were treated with EMR/ESD. There were no cancers in this group. None of them were treated with TEM or ESD. Three percent had a history of segmental bowel resection for a benign disease (Table 2).

#### 3.2.4. Cancer Follow-Up

All the patients followed after treatment for a colorectal cancer had undergone a segmental bowel resection. Fifteen percent had adenomas (*n* = 37/251). Thirty-one were treated with a snare resection, and 3 with an advanced endoscopic resection. One cancer was found, and 3 adenomas were left untreated (Table 2).

#### 3.2.5. Increased Risk of Hereditary Cancer

There were 96 patients with a history of hereditary colorectal cancer. 34 positive colonoscopies were registered; of which, 30 were treated with a simple snare resection. One was referred to colectomy for a cancer treatment. Fifty-six percent had a simple snare resection of an adenoma earlier, and 33% of them had a new adenoma on a surveillance colonoscopy (Table 2).

## 4. Discussion

Almost one third of the patients referred to the colonoscopy had a significant adenoma. The diagnostic yield varied depending on the indication for referral. The highest adenoma rate was seen in patients followed after an endoscopic resection of a benign adenoma, leading to adenoma detection in almost half of the cases. The patients earlier were treated for a malignant colorectal tumor, and the screening individuals revealed the lowest frequency of adenomas and cancers.

There is a variety of symptoms leading to the referral of patients for a colonoscopy, making this group the most inhomogeneous one in respect to the indications for a colonoscopy. Nevertheless, this group showed a rather high adenoma rate of 25%, indicating that the clinical judgement made by a physician is a strong predictor for pathological findings as a screening test or a surveillance colonoscopy. Of the 250 colonoscopies in the group with symptoms, 207 were first-time colonoscopies yielding even more polyps where 63 colonoscopies were positive, resulting in 30% with significant findings. Seven had a segmental bowel resection for causes other than cancer.

The adenoma prevalence after a positive screening test is lower compared to other studies especially in the UK and The Netherlands, and it is also lower than that of the Danish National Screening Database. Our unit has a high adenoma detection rate compared to other national centers (sundhed.dk, dts aarsrapport, January 2016, https://www.sundhed.dk/sundhedsfaglig/kvalitet/kliniske-kvalitetsdatabaser/screening/dansk-tarmkraeftscreeningsdatabase/, cited 2016 March), and the low frequency of screen-detected adenomas in this population is most likely incidental. The adenoma diagnostic yield of the iFOBT-positive individuals was almost in every fifth patient; the vast majority of the adenomas were resected with a simple snare. The diagnostic yield of the follow-up colonoscopy, after earlier resection of a benign adenoma, was showed to be high. Almost half of the patients had adenomas. Most of them were resected with a snare, but it also revealed the highest number of adenomas referred to advanced endoscopic resections. Forty-four percent of patients with previous simple snare resections had a new adenoma. Another noteworthy point in this group is the high number of adenomas found.

The lowest diagnostic yield seems to be after a resection for malignancy. Only 15% of those had an adenoma. All except 1 patient were treated with a simple snare resection, and only 1 cancer was found. One could consider performing the follow-up colonoscopy with larger intervals, which has already been determined in Denmark. Another obvious explanation for the rare findings in this group is that they have had a segment of their bowel removed, making the probability of new pathology smaller.

As seen in the group with a family history, the surveillance colonoscopies ensure that the tumors are found in proper time, yielding only 1 cancer and, otherwise, adenomas managed by a simple snare resection, even though 58% of the patients in this subgroup had adenomas and therefore a high diagnostic yield of adenomas from a surveillance colonoscopy. We perform 9000 colonoscopies a year in our unit. If our expert endoscopists trained in EMR and ESD were doing the index colonoscopy in the groups with the highest risk of large adenomas, we could save approximately 360 patients yearly for a second colonoscopy.

## 5. Conclusion

Our study indicates that patients who had an earlier endoscopic resection for an adenoma and patients with a family history of hereditary colorectal cancer have the highest diagnostic yield from surveillance colonoscopy. We found that symptoms are strong predictors for positive findings. A high frequency of patients was treated with a simple snare resection. Attention should be payed to patients who had a resection of an adenoma, because of their risk of developing new adenomas, and a higher risk of referral to advanced endoscopic treatment.

## Figures and Tables

**Table 1:** 

	Indication group^∗^					
	1	2	3	4	5	Total
Patients enrolled	246	251	201	242	59	999
Median age (years)	63.8 (±12.9)	64.8 (±8.0)	66.7 (±8.9)	70.1 (±10.5)	52.1 (±17.7)	65.1 (±12.0)
Sex, M/F	129/121	143/108	154/96	131/120	44/52	601/497
Colonoscopies	250	251	250	251	96	1098
Positive colonoscopies (%)	63 (25.2)	42 (16.7)	113 (45.2)	37 (14.7)	34 (35.4)	289 (26.3)
Number of adenomas found^∗∗^	117	107	254	63	50	591
Colonoscopies with simple snare resection (%)	47 (18.8)	38 (15.1)	103 (41.2)	29 (11.6)	30 (31.3)	247 (22.5)
Referral to advanced endoscopic resection (EMR/ESD/TEMS)	17 (6.8)	10 (4.0)	16 (6.4)	1 (0.4)	1 (1.0)	45 (4.1)
Cancers found	2	3	0	1	1	7

^∗^The numerals act as synonyms for the indication groups. 1 = symptoms. 2 = positive screening for CRC cancer. 3 = surveillance after treatment of benign polyp. 4 = surveillance after treatment of colorectal cancer. 5 = surveillance of patients with high risk of developing hereditary colorectal cancer. ^∗∗^Colonoscopies resulting in an uncountable number of polyps are not included. There were six colonoscopies with an uncountable number of polyps, five cases in group 5 and 1 case in group 1.

**Table 2:** Colonoscopy findings stratified by previous findings.

	Indication group^∗^											
	1		2		3		4		5		Total	
	Previous^∗∗^	Positive^∗∗∗^	Previous^∗∗^	Positive^∗∗∗^	Previous^∗∗^	Positive^∗∗∗^	Previous^∗∗^	Positive^∗∗∗^	Previous^∗∗^	Positive^∗∗∗^	Previous^∗∗^	Positive^∗∗∗^
No previous colonoscopy	207	56	209	38	0	0	0	0	14	9	430	103
Previous normal colonoscopy	24	2	26	2	0	0	0	0	20	5	70	9
Previous simple polypectomy	12	4	14	2	194	86	0	0	54	18	274	110
Previous advanced polypectomy	0	0	0	0	53	24	0	0	0	0	53	24
Segment bowel resection	7	1	2	0	3	3	251	37	8	2	271	47
Total	250	63	251	42	250	113	251	37	96	34	1098	293

^∗^The numerals act as synonyms for the indication groups. 1 = symptoms. 2 = positive screening for CRC cancer. 3 = surveillance after treatment of benign polyp. 4 = surveillance after treatment of colorectal cancer. 5 = surveillance of patients with high risk of developing hereditary colorectal cancer. ^∗∗^Previous intervention. This column shows what procedures patients of this indication group previously had. ^∗∗∗^Positive colonoscopy. This column lists the patients with a present positive colonoscopy stratified by previous intervention they have had.
